# Antibody Screening by Microarray Technology—Direct Identification of Selective High-Affinity Clones

**DOI:** 10.3390/antib9010001

**Published:** 2020-01-02

**Authors:** Martin Paul, Michael G. Weller

**Affiliations:** Federal Institute for Materials Research and Testing (BAM), Division 1.5 Protein Analysis, Richard-Willstätter-Strasse 11, 12489 Berlin, Germany; martin.paul@bam.de

**Keywords:** monoclonal antibodies, mabs, fusion, false positives, hapten immunoassays, competitive immunoassays, ELISA, antibody validation, antibody quality, microarray, hybridoma technology, linker recognition, high-throughput screening, HTS, heterology concept

## Abstract

The primary screening of hybridoma cells is a time-critical and laborious step during the development of monoclonal antibodies. Often, critical errors occur in this phase, which supports the notion that the generation of monoclonal antibodies with hybridoma technology is difficult to control and hence, a risky venture. We think that it is crucial to improve the screening process to eliminate most of the critical deficits of the conventional approach. With this new microarray-based procedure, several advances could be achieved: Selectivity for excellent binders, high-throughput, reproducible signals, avoidance of misleading avidity (multivalency) effects, and performance of simultaneous competition experiments. The latter can also be used to select clones of desired cross-reactivity properties. In this paper, a model system with two excellent clones against carbamazepine, two weak clones, and blank supernatant containing fetal bovine serum was designed to examine the effectiveness of the new system. The excellent clones could be detected largely independent of the immunoglobulin G (IgG) concentration, which is usually unknown during the clone screening since the determination and subsequent adjustment of the antibody concentration are not feasible in most cases. Furthermore, in this approach, the enrichment, isolation, and purification of IgG for characterization is not necessary. Raw cell culture supernatant can be used directly, even when fetal calf serum (FCS) or other complex media is used. In addition, an improved method for the oriented antibody-immobilization on epoxy-silanized slides is presented. Based on the results of this model system with simulated hybridoma supernatants, we conclude that this approach should be preferable to most other protocols leading to many false positives, causing expensive and lengthy elimination steps to weed out the poor clones.

## 1. Introduction

During antibody development, the screening of hybridoma cells is a crucial step. Several obstacles may lead to a complete failure of the process. First, the assay needs to be selective (“specific”) enough. Otherwise, the researcher is flooded with seemingly positive clones, which, in a later stage, turn out to be of poor quality or completely negative. Good clones might be irreversibly lost in this phase because, in most cases, it is not feasible for all clones to undergo an in-depth examination. The second requirement is speed since some irrelevant hybridoma cells might grow very fast and overgrow some positive clones, if the final clonal state is not yet reached. The third point is parallelization, since the probability that an excellent clone is found increases with the number of clones tested. Due to technical and financial limitations, often, too few clones are examined. The fourth issue is mainly encountered with haptens. Quite often, antibodies, which bind to an immobilized hapten–protein conjugate and hence are identified as positives, are found to be weak or non-binders of the free analyte. This effect is known as spacer recognition, linker recognition, or the bridging phenomenon [1,2,3,4,5,6,7,8,9,10,11,12,13]. Sometimes, this effect can be reduced by the application of linker or site heterology using different linkers or conjugation reagents for the preparation of immunogens and coating antigens or enzyme conjugates [14,15]. The fifth point refers to the affinity of the respective clones. In most cases, an affinity ranking is required to identify the strongest binder, which often shows the best performance in analytical or diagnostic applications [16] in contrast to therapeutic antibodies, where a subtle balance between pharmacokinetics, efficient penetration, and retention in the target tissue needs to be achieved [17,18]. The sixth risk factor is the screening date. Since the antibody screening is a cost and time-intense step, the procedure is usually performed only once or twice. However, due to the varying growth rate of individual hybridoma clones, the “best” time for screening can hardly be determined. Consequentially, several rounds are necessary to catch both fast and slow cells. The fast cells need to be ranked immediately since they might be lost on a later date.

Most popular screening procedures show one or several of these drawbacks and therefore increase the risk of unsatisfactory antibody development. This might be the reason why such projects are still high-risk endeavors, which is particularly unpleasant when project partners or customers are critically dependent on timely antibody deployment.

Here, we present a novel screening format, which should be highly favorable for most projects based on hybridoma technology and conveniently feasible for most laboratories. This protocol overcomes the hurdles mentioned above and is based on microarrays performed on a standard slide format. The first important advancement is the use of an antibody-immobilized format, which in this context has been proposed in [19], in contrast to antigen-immobilized formats, which are recommended in most textbooks and articles. The second improvement is the miniaturization of the assay, which is achieved by the use of a microarray format, which has been used favorably in many applications, e.g., [20,21,22,23,24]. This enables the fast and easy performance of screening, sometimes with only a single chip. However, the third feature might be the most innovative in this context. The microarray-based test can be performed in a true competitive format, which leads to both the identification of true positives and the affinity ranking of the clones. Even some basic cross-reactivity tests might be possible. We have performed a model screening with known clones, which had been identified and characterized previously [25,26].

In the context of hybridoma technology, microarray-based screening formats have been presented in several publications [21,27,28,29,30,31,32,33,34,35,36]. Nearly all used antigen-immobilized formats have all of the limitations mentioned above. Due to their fundamentally different approaches, we do not discuss them in more detail here. Most of these protocols have found only very limited applications until today, and as a consequence, a practical microarray approach for antibody screening is still lacking.

Carbamazepine (CBZ) is an important antiepileptic drug, which is prescribed frequently and at a relatively high dose. Due to its poor degradability, it is found in many surface and ground waters and, therefore, can be used as an anthropogenic contamination marker. Several immunoassays have been developed for the detection of CBZ, which require the availability of suitable antibodies, which are nearly always a limiting resource in immunochemical applications. Recently, new antibodies against CBZ have been developed in our department [25,26]. We used some of these clones as model antibodies for the setup and optimization of a novel chip-based hybridoma screening procedure, which is presented here as a feasibility study. The application of this approach to complete projects for antibody development is planned to be performed and published in the future.

## 2. Materials and Methods

### 2.1. Reagents, Buffers, Materials, and Equipment

Transparent, flat-bottomed non-binding 96 microwell plates were acquired from Greiner Bio-One (Frickenhausen, Germany), PD SpinTrap™ G-25 Desalting Columns were obtained from GE Healthcare (Uppsala, Sweden), and clear microscope slides were bought from Sigma-Aldrich (Darmstadt, Germany). Recombinant Protein G (PRO-402) and Cys-Protein-G (PRO-1238) were purchased from Prospec (Ness-Ziona, Israel), monoclonal anti-CBZ antibody BAM-mab 01 (CBZ) was obtained from BAM (Federal Institute for Materials Research and Testing (BAM), Berlin), anti-CBZ antibody B3212M (Meridian Life Science Inc, Memphis, USA) was kindly supplied by S. Flemig (BAM), and the clones 3B3 and 6C5 were supplied by M. Dippong [26] (BAM). Fetal calf serum (Biochrom S0115), l-glutamine, RPMI1640, and 2-mercaptoethanol were acquired from Biochrom (Berlin, Germany). The fluorescence dyes Dy654-NHS and Dy554-NHS were purchased from Dyomics (Jena, Germany). According to the manufacturer, the following properties of the dye Dy654 are given: Absorption/emission maximum: 653/677 nm (in ethanol); molar absorbance: 220.000 M^−1^ cm^−1^; and soluble in water, methanol, and dimethylformamide (DMF). The mono-protected PEG3 linker 1-(9-fluorenylmethyloxycarbonyl-amino)-4,7,10-trioxa-13-tridecanamine hydrochloride (Fmoc-TOTA·HCl) was bought from Iris-Biotech (Marktredwitz, Germany). Carbamazepine (CBZ), Dibenz[b,f]azepin-5-carbonyl chloride (CBZ-Cl, Sigma, 90 %), bovine serum albumin (BSA, Sigma, >98%) and (3-glycidyloxypropyl)trimethoxysilane (Glymo, Sigma, >98%), DMSO (AppliChem, >99.5%), glycerol (Sigma-Aldrich G2025), Tween 20 (Sigma, P7949), hydrochloric acid (Fluka, 84415), sodium hydroxide (Sigma, 30620), Mucasol (Sigma, Z637203), tetrahydrofuran (THF, Chemsolute, >99.9%), and toluene (Roth, >99.5%) were obtained from Sigma-Aldrich. Ultrapure water (MilliQ) was supplied by a Milli-Q Synthesis A10 (Merck, Germany). Cyano-4-hydroxycinnamic acid was bought from Bruker (201344). Nexterion E slides (Epoxy) were a sample from Schott.

The washing buffer was made of 10 mM phosphate, 150 mM sodium chloride, and 0.05 vol% of Tween 20 (PBST005, adjusted to pH 7.4). The spotting buffer PBSGT (10 × PBS, adjusted to pH 8.0) contained 100 mM of sodium hydrogen phosphate, 1500 mM of sodium chloride, 2.5 vol% of glycerol, and 0.00625 vol% of Tween 20. The cell culture medium (CCM) was prepared from 270 mL of RPMI 1640 (Biochrom F1215), 30 mL of fetal bovine serum (S0115 Superior, 0439X), 3 mL of 200 mM L-glutamine (Biochrom L0282), and 300 µL of 2-mercaptoethanol (Biochrome M3148). Cell culture medium with glycerol and Tween 20 for spotting (CCMGT) was prepared by supplying CCM 24:1 with 50 vol% glycerol containing 0.125 vol% Tween 20.

The spotting was carried out with a BioOdyssey Calligrapher Miniarrayer (BioRad Laboratories, München, Germany) equipped with MCP310S solid pins (spot diameter about 400 µm, volume approximately 1 nL, BioRad Laboratories). The glass slides were scanned with a DITABIS Microarray Scanner MArS (Pforzheim, Germany) using the red/green filter set in the 10-µm fast scanning mode. MALDI-TOF MS was performed with a Bruker Autoflex II Smartbeam mass spectrometer.

### 2.2. Preparation of Epoxy Slides

Transparent glass slides (25 × 75 × 1 mm) were sonicated for 15 min at room temperature (RT) in a 2 vol% solution of Mucasol universal detergent, rinsed with pure water and etched for one hour in sodium hydroxide solution (10%), and rinsed with pure water. The etched slides were treated in 37% hydrochloric acid for 2 h, washed with pure water, and dried by placing the slides in a gentle airstream. Then, 1 vol% of water was added to toluene and stirred for 5 min. Subsequently, 1 vol% of Glymo was added and stirred for another five minutes. The slides were incubated in this solution for 18 h at RT. Subsequently, the slides were washed with isopropanol and pure water. After the silanization, the slides were highly hydrophobic. The epoxy slides can be stored for a longer time in a dry containment at RT.

In the first step, the epoxy slides were spotted by Cys-Protein-G. The printing solution consisted of Cys-Protein-G (1 g/L) diluted 1:5 in PBSGT (pH 8.0). The spotting procedure was performed at 65% humidity and 15 °C using MCP360S pins with a 400-µm diameter, transferring approximately 1 nL of the solution per spot, resulting in a 12 × 8 spot array with a 1000-µm spot-to-spot distance. The printed slide was incubated for three days in an airtight 50-mL falcon tube over PBS with 1% glycerol in the dark. After the incubation, the slide was washed with PBST, purged with 0.1 vol% of glycerol, dried with nitrogen, and directly used for the screening experiments. No further blocking steps were applied to the chip. These protein G chips can be stored cool or frozen in the dark for future projects.

### 2.3. Sample Printing and Incubation

In the next step, the immobilization of the antibodies from cell culture supernatants was examined. As a model system, we used a typical cell culture medium supplied with 10% of fetal calf serum, spiked with the respective antibodies (see Table 1) at different concentration levels ranging from 0.1 to 10 mg/L. The transfer from the 96-well source plate (non-binding, Greiner Bio-One, 655901, F-Bottom) to the microarray was performed with a Calligrapher™ MiniArrayer (BioRad). For the printing step, MCP360S solid ceramic pins were used, which transferred approximately 1 nL of the sample. Thus, only extremely small amounts of supernatant were consumed, and a nearly unlimited number of replicates could be performed, if required. The samples were reprinted in a 12 × 8 subgrid at the very same coordinates on which the Cys-Protein-G was previously immobilized. For some assays (e.g., inhibition, see below), replicates are performed on the same chip. After 18 h of incubation at 4 °C, the chip was washed thoroughly again. It should be considered that no extended washing steps of the ceramic pin were performed in the spotting procedure, due to time considerations. This and some other washing issues may lead to some carryover in rare cases. Nevertheless, these effects can easily be identified and corrected during data evaluation.

### 2.4. Design and Synthesis of Hapten–Fluorophore Conjugates

**Figure 1:**
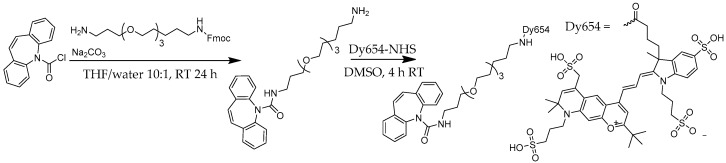
Synthesis of the CBZ-TOTA-Dy654 tracer [26].

The screening procedure relies on fluorophore-labeled antigens or haptens. The proper choice of the dye is of considerable relevance. Today, highly advanced fluorescence labels are available, which display many desirable properties, like a high quantum yield, high photostability, excellent water solubility, reduced aggregation, and low non-specific binding. Based on the available laser excitation source of 635 nm, the dye Dy654 (Figure 1) was chosen.

First, CBZ-TOTA-amine was synthesized by a nucleophilic substitution reaction of dibenz[b,f]azepine-5-carbonyl chloride with a semi-protected Fmoc-TOTA spacer. The Fmoc group was subsequently cleaved under mildly basic conditions, and the unprotected terminal amino group was reacted with an equimolar amount of NHS-activated Dy654 in DMSO as described in [26]. The conjugate was used without further purification.

### 2.5. Competition Experiments

**Figure 2:**
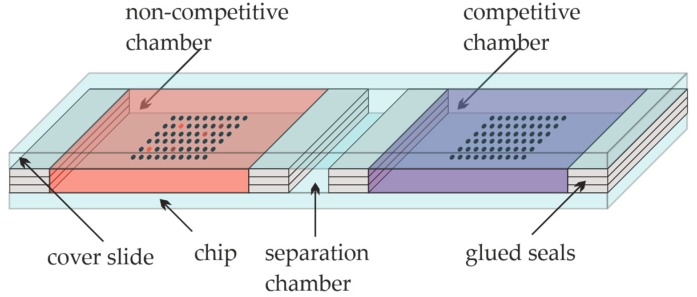
Competition experiment on a microarray slide.

The glass slides were epoxy-functionalized, coated with Cys-Protein-G sub grids (12 × 8 spots), and respotted with the sample solutions as described above. For the competition experiments, the slide was divided into three different areas with glued seals. Four seals manufactured of three vertical stacks of laboratory adhesive tape (Tough-Tags^TM^) were glued onto the slide, and a blank slide was placed on top of them. In this way, three separated chambers, the “non-competitive” and the “competitive” one, with a separation chamber in between, were created, as shown in Figure 2. Each of the main chambers had dimensions of approximately 25 mm × 20 mm × 0.3 mm. The first cavity, the non-competitive cavity of the slide, was filled with approximately 150 µL of diluted CBZ-TOTA-Dy654 tracer in PBST (1:10.000), the separation cavity was kept empty, while the third cavity, the competitive chamber, was incubated with approximately 150 µL of CBZ-TOTA-Dy654 tracer in PBST (1:10.000) with the addition of 26 mg L-1 of CBZ. The incubation was performed simultaneously in both cavities for one hour in the dark at RT. Subsequently, the cover slide was removed, and the microarray was rinsed with PBST and 0.1 vol% of glycerol and dried quickly with nitrogen. The final washing steps after the tracer incubation required approximately 1 min in total. The slide was scanned with the microarray scanner at 100 % PMT (photomultiplier) intensity in the 10-µm fast scanning mode. For the 635-nm excitation, the red filter was used.

The scan of the whole slide consisting of a 16-bit TIF file was imported in Fiji-ImageJ software (V.2.0.0-rc-43) (National Institutes of Health and the Laboratory for Optical and Computational Instrumentation (LOCI, University of Wisconsin), source: https://imagej.nih.gov/ij/download.html) [46,47], corrected for angular misalignment, and cropped into two separate files: The non-competitive and the competitive array. Each array was saved individually as a 16-bit TIF file without any additional preprocessing applied to the raw data. For each crop file, the center *X*- and *Y*-coordinates of the upper left spot were determined for further semi-automated data evaluation.

## 3. Results

### 3.1. The Coating of Microarray Slides

Surface chemistry is a crucial point for microarrays. Epoxy-silanized glass slides were chosen here because they have been proven to show excellent performance in antibody applications [48,49]. In a first step, they were coated with protein G [50], which enables a very efficient and oriented immobilization [51] of most immunoglobulin classes. Finally, a protein G coating is expected to enrich antibodies from the complex cell-culture supernatants due to the selective interaction between protein G and immunoglobulins. For the repeated use of the same antibody-coated chip, a novel preactivation crosslinking may be used [52]. However, this advanced protocol was not applied in this work, yet.

**Figure 3:**
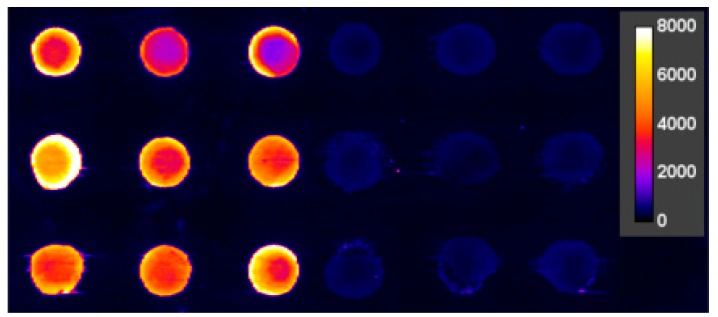
Comparison of different surface coatings for antibody immobilization. In this experiment, fluorophore-labeled goat IgG was incubated on spots of immobilized Cys-Protein-G (**left**) and protein G (**right**). The spot to spot distance is 1 mm in the *x*- and *y*-dimension.

It could be shown in preliminary tests that a cysteine-modified protein-G, Cys-Protein-G (Figure 3), consistently showed a higher immobilization efficiency for IgG, which supports the notion that the additional cysteine leads to an improved immobilization on epoxy slides at a pH 7 and 8. Previously, Cys-Protein-G was mainly used on gold surfaces [53,54], on which the strong thiol–gold interaction leads to an oriented and efficient immobilization. Since gold-coated slides are quite expensive, we preferred epoxy-silanized surfaces on conventional glass slides for our screening approach. In experiments with epoxy-functionalized glass substrates, spotted Cys-Protein-G showed significantly higher fluorescence signals for fluorescently labeled goat IgG (Figure 3). Therefore, in further experiments, Cys-Protein-G was used exclusively. The selective pre-spotting of the chip with Cys-Protein-G instead of pre-coating the whole chip with this reagent has the advantage of well-defined spot shapes and a significantly reduced consumption of Cys-Protein-G.

### 3.2. Antibody Printing

In the next step, the immobilization of the antibodies from protein-rich cell culture supernatants was examined. As a model system, we used a typical cell culture medium supplied with 10% of fetal calf serum with glycerol and Tween 20 to improve the spot shape, spiked with the respective antibodies at different concentration levels. The transfer with the contact spotter transferred approximately 1 nL of the sample volume on the chip. Hence, only extremely small amounts of the supernatant were consumed, and a nearly unlimited number of replicates could be performed, if required. The spots were reprinted in a 12 × 8 subgrid on the same coordinates on which the chip had been coated with Cys-Protein-G to allow for antibody immobilization.

### 3.3. Design and Synthesis of Hapten–Fluorophore Conjugates

**Figure 4:**
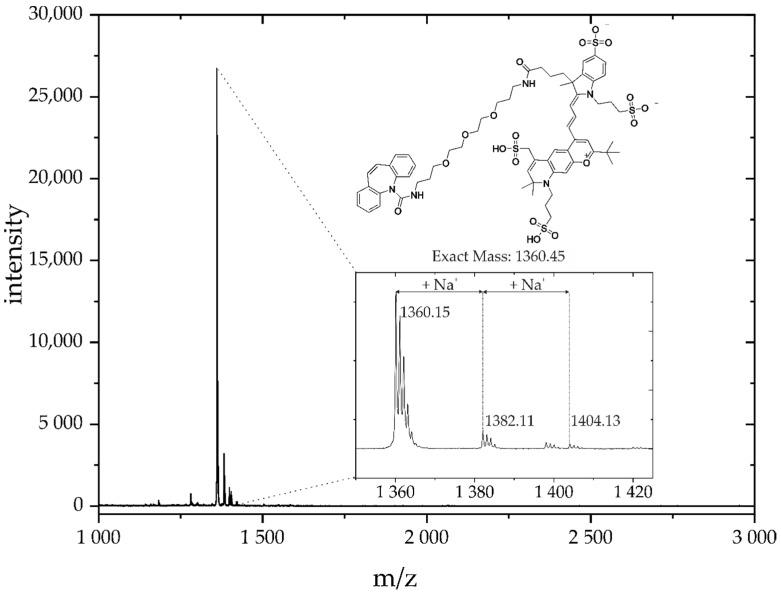
MALDI-TOF-MS analysis of the carbamazepine-Dy654 conjugate. The expected molecular mass of the compound in the negative mode is 136,045. Due to the various sulfonic acids, the molecule is prone to exhibit sodium adduct peaks.

Based on the available laser excitation source of the scanner (635 nm), and due to the high hydrophily, the dye Dy654 (Figure 1) was chosen. To avoid steric hindrance and unwanted interaction between hapten (immunoreactive group) and fluorescent dye, a short polyethylene glycol linker (TOTA) was used. The CBZ-TOTA-Dy654 tracer was synthesized in two steps [26] and examined by matrix-assisted laser desorption/ionization time-of-flight mass spectrometry (MALDI-TOF-MS) (Figure 4). The tracer showed no unspecific interaction with epoxy-silanized glass slides.

### 3.4. Incubation Steps of Reagents and Hybridoma Supernatants

**Figure 5:**
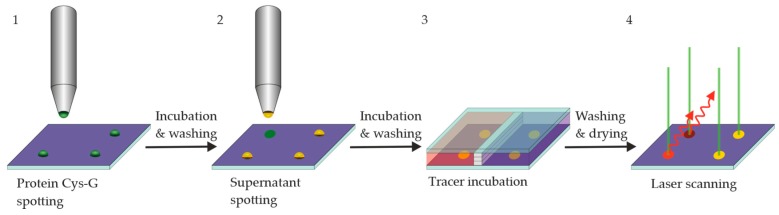
A general approach for the chip-based screening: 1. Printing of Cys-Protein-G on an epoxy-silanized glass slide (may be prepared in advance); 2. Printing of mAb supernatants; 3. Incubation of labeled antigen/hapten. In separate chamber(s), but on the same slide, competition experiments can be performed; 4. Laser scan to quantify fluorescence signals.

All immunochemical steps were performed on epoxy-silanized glass slides (Figure 5). In a first layer, Cys-Protein-G was printed on the slide with 400-µm pins in a 1000-µm grid. After incubation for three days, the slides were washed and dried; these slides could be used directly or stored for future projects.

**Figure 6:**
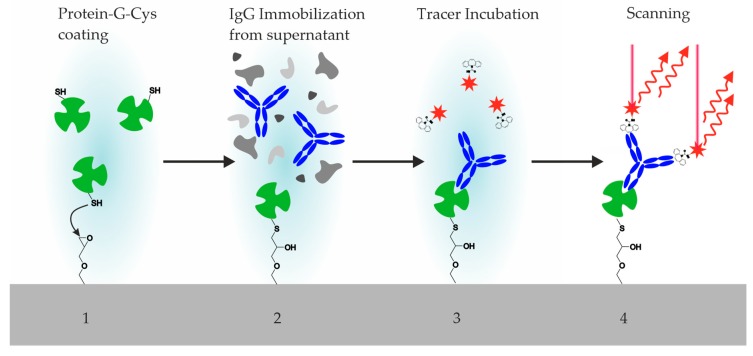
Incubation steps of the hybridoma screening process: 1. Printing and incubation of an epoxy-silanized glass chip with a Cys-Protein-G solution. Washing step. 2. Printing and incubation of hybridoma supernatant. Washing step. 3. Incubation of fluorescence tracer (labeled antigen or hapten). Washing step. Drying. 4. Fluorescence scan (Exc. 635 nm, Em. 650–670 nm). (5. Regeneration is optional).

For the simulated screening process, raw hybridoma supernatants (here, model solutions of known clones) were stored in a 96-well microwell plate. The simulated hybridoma supernatants were transferred from this source plate on to the Cys-Protein-G spots on the previously prepared chip. For some assays (e.g., inhibition), replicates are prepared on the same chip. After the incubation, the chip was washed again and subsequently incubated with the fluorescence tracer CBZ-TOTA-Dy654. After a short washing step, the chip was dried and examined by a conventional fluorescence scanner (Figure 6).

The assay type described above was rarely applied in the literature. It shows some distinct advantages:

(a) The dye conjugate is monovalent, which avoids confusing avidity (multivalency) effects, which are often misinterpreted.
(b) This monovalent binding restricts the signals to high-affinity antibodies. With weakly binding antibodies, the tracer (labeled antigen) is washed away. The washing duration might modulate the cutoff of the detected clones according to their off rate.
(c) The tracer binding is highly reversible, which makes it possible to reuse the chip without strong regeneration steps.

Multivalency is also a frequent problem in surface-plasmon resonance (SPR) measurements, which are often used for antibody characterization. Unperceived multivalency leads to misleadingly high affinity constants, overestimating the quality of an antibody [55,56,57,58].

### 3.5. Data Evaluation

**Figure 7:**
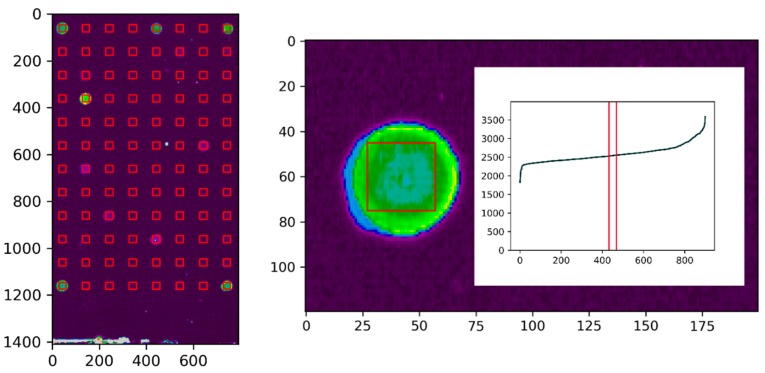
Scan of the microarray (**left**) with generated square regions of interest (ROI) over all spots of the array for the semi-automatized data evaluation with the Python script. ROI of the first spot (**right**) with included pixels, which were sorted according to their intensity. In total, 2% of the central pixels were used for the evaluation.

In the semi-automated data evaluation with Python (Anaconda Spyder 3.3.2), the previously saved non-competitive and the competitive crop-files were imported, and as manual input, the *x*–*y* start coordinates along with basic grid parameters were entered. In the first step of the data evaluation by the script on the center of every spot in the array, a square region of interest (ROI) of 30 × 30 pixels was defined, see Figure 7.

For every individual ROI, all included pixels were sorted according to their intensities. The central 2% of the pixels were used to calculate the truncated mean of the spot intensity, and the remaining pixels in the ROI were trimmed (truncated) in order to achieve a highly robust estimate of the mean. This accounted for even severe spot inhomogeneities and significantly increased the robustness of the spot evaluation. The procedure was performed simultaneously for the non-competitive and the competitive array. Subsequently, the quotient of the corresponding non-competitive and competitive spot was calculated and stored in a table. This quotient was used to assess the quality of the clones. A high quotient translates to a clone with a high affinity for the fluorescent tracer, as the tracer was strongly bound by the captured IgG from the supernatant. Simultaneously, a high quotient shows a successful competition with the target analyte, as the analyte inhibited the binding of the tracer. Finally, the spot intensities, along with the quotient, were exported as a txt file by the script.

### 3.6. Identification and Ranking of Hybridoma Clones

As a model system, we used three positive clones of previous hybridoma projects for the development of improved carbamazepine (CBZ) antibodies and one commercial clone of proven quality. The monoclonal antibodies possessed quite different affinities against their target; they coverd about four decades (Table 1). These clones have been described or used in previous publications [25,26,37,39]. Dots of a cell culture medium were used as negative controls. One of the most critical points of such hybridoma screenings is the influence of the unknown IgG concentration. In theory, it might be possible to determine the IgG concentration independently and to dilute the supernatants accordingly. However, this approach seems to be quite impractical and hence not useful. Therefore, we tried to get along with varying antibody concentrations and tested three different levels of 0.1, 1, and 10 mg/L.

**Figure 8:**
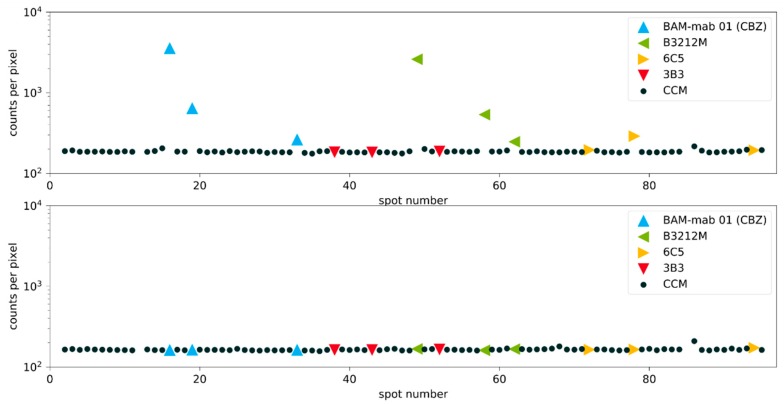
Clone screening with inhibition experiment. Signals for the spots on the Cys-Protein-G glass slide. The upper figure shows the non-competitive binding of the fluorescently labeled hapten (analyte) to the immobilized antibodies. The lower figure shows the same experiment under competitive conditions with an excess of hapten (27 mg L^−1^ analyte CBZ). Known monoclonal antibodies diluted in cell culture medium are color-coded, accordingly. CCM: Cell culture medium (negative controls). Blank measurements with slightly increased signals are caused by carryover effects, which can be easily identified and eliminated.

In Figure 8, the normalized fluorescence signals of 92 samples are shown ordered by spot number. In typical screenings, the vast majority of all tested clones show no affinity for the hapten and therefore signals on the background level are available in abundance.

**Figure 9:**
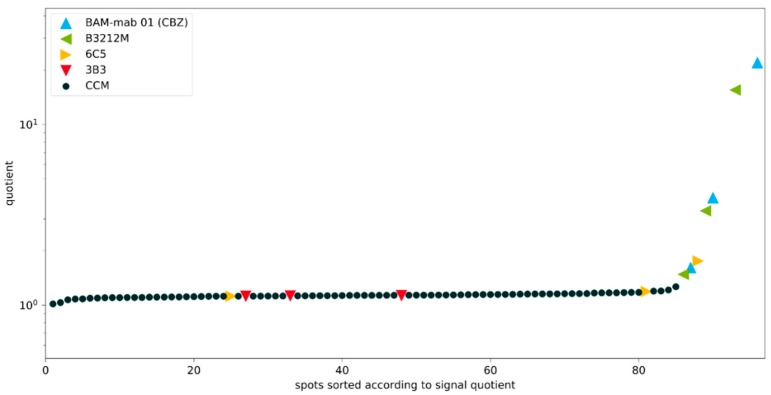
Clone screening with inhibition experiment (quotient, sorted). Signal quotients of the non-competitive and the competitive spots for the spots on the Cys-Protein-G glass slide. Known monoclonal antibodies diluted in cell culture medium are color-coded, accordingly. CCM: Cell culture medium (negative controls). The good antibodies BAM-mab 01 (CBZ) and B3212M are easily identified. With a cut-off value of 2 for the quotient, the weak antibody 6C5 is always below this value, even at high concentrations, and the poorest antibody 3B3 is not different from the blank values. It is noteworthy that 3B3 could not be clearly distinguished from high-affinity clones in an original publication (Figure 3 in [26]).

Although a small drift of the signal was found, the signal quotient is quite stable. Most important, however, is the unambiguous identification of the high-affinity clones B3212M and BAM-mab 01 (CBZ). The quotients at all concentrations (0.1, 1, and 10 mg/L) were significantly above the negative controls. These excellent clones would have been identified under any circumstance. This is the most important finding of this work. However, also, false positives need to be minimized. In theory, many other screening protocols can identify good clones, but they generate such a flood of false positives, leading to the unfortunate situation that some or all of the good clones may be lost. Figure 9 also shows that this method is not distracted by poor clones. Very weak antibodies go completely undetected as the clone 3B3 (red triangles), or they are only slightly positive at very high concentrations (yellow triangles), but still remain below the cut-off value of 2. Nevertheless, from our point of view, the most practical way to choose positive clones is to avoid any (arbitrary) cut-off values, but simply to start with the highest quotient, collect more clones in the direction from the highest to the lowest quotient and stop when there is a sufficient number of clones, or there are no more clones significantly different from the background. The model screening on the inhouse manufactured epoxy-chips was reproduced on commercially available epoxy-silanized microarray slides (see Supplementary Materials). Essentially, the same results were obtained. This supports the notion that the novel process is highly reproducible in contrast to other protocols, which often lead to quite variable and ambiguous outcomes.

### 3.7. Competition Experiments

**Figure 10:**
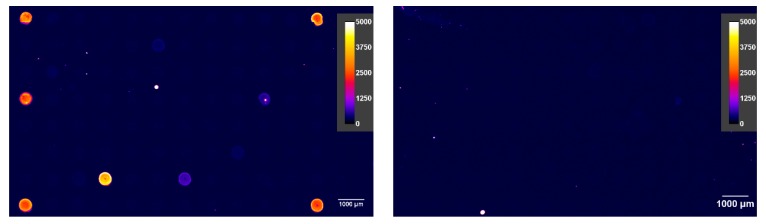
Non-competitive mAb incubation (**left**) and the competitive incubation with 27 mg L^−1^ carbamazepine (CBZ) (**right**). The positive spots are strongly inhibited by the hapten (analyte), which means that the respective antibodies bind selectively to the target compound, CBZ.

Essentially, all screening protocols published before did not use competitive assays for the primary examination of hybridoma clones. In contrast, the direct (antibody-immobilized) format used in this work avoids avidity effects and efficiently suppresses other unwanted false positives, sometimes loosely termed “linker recognition”. Competitive formats provide additional evidence for the performance of the respective clone in competitive assays. This approach directly rejects all clones, which cannot be inhibited by the target analyte at a user-defined concentration (Figure 10). However, this format is even more powerful. It enables the user to examine cross-reactivity properties at a very early stage of clone screening and thus to pick the best clones for a respective application. Considering the opportunity to regenerate the slides, the cross-reactivity experiments may be repeated several times to check all cross-reactants of interest. It is not necessary to postpone this characterization and selection to the time after clone expansion. Due to the small spots and hence the density of the arrays, several replicates of the clones can be printed on one slide. Using incubation chambers with not only two but several separated wells, parallel incubations without and with different competitors may be performed in one run. The strict focus on the best clones and the rejection of all non-binding and non-inhibited clones saves a lot of time and money, since the recloning, expansion, antibody isolation, and purification are by far the most expensive and time-consuming steps in the development of monoclonal antibodies, which have to be performed with each seemingly positive clone.

## 4. Discussion

The development of monoclonal antibodies is still a risky and expensive endeavor. Inefficient and error-prone screening procedures cause unnecessary costs and project delays. We are convinced that poor antibody clones should be eliminated in the development process as early as possible. Unfortunately, nearly all textbook protocols rely on screening steps of limited selectivity. More powerful validation steps are often shifted to later stages of the project, after labor and cost-intensive steps like recloning and the expansion of many seemingly positive clones have been performed. In addition, for many characterization techniques, purified antibodies are required. From this retrospective point of view, it is often recognized that the selected clones are of disappointing quality or even negative. Our approach uses several measures to improve this situation: First of all, the miniaturization of the process enables the testing of a very high number of clones, which avoids that clones are lost due to arbitrary pre-selection criteria or other limitations. The next improvement is based on the use of an antibody-immobilized format, which is not yet routinely used. This leads to the suppression of unrecognized avidity effects, which often cause the overestimation of the affinity of poor antibody clones. After efficient washing steps, only strong binders with low inhibition concentrations are detected as true positives. Therefore, this protocol is particularly useful when antibodies with the highest affinity are desired since clones with low affinity will not be detected. The functional affinity cut-off might first be influenced by the stringency of the washing step and second by the concentration of the competitor(s). Finally, the parallel performance of inhibition assays confirms the selectivity and the focus on the right target. Otherwise, many antibodies bind strongly to the immunogen conjugates, but not or only weakly to the intended target. In addition, even a more complex inhibition screening might be performed, if very special cross-reactivity restrictions must be met. Nevertheless, perhaps the most relevant advantage of this novel approach for hybridoma screenings might be the cost-cutting and time-saving effect by discarding many poor clones in the very first screening step.

## 5. Conclusions

With the aid of simulated hybridoma supernatants based on known monoclonal antibodies, it could be shown that the presented approach is extremely efficient in identifying high-affinity clones, with essentially no false positives. We plan to apply this protocol to our upcoming antibody projects to reduce the costs and the time efforts for the development of high-quality antibodies, which are desperately needed in many bioanalytical ventures [16,59].

## Figures and Tables

**Table 1:** Monoclonal antibodies used for the simulated screening.

Antibody	IC_50_ (µg/L)	Isotype	Rating	Test Result (1 mg/L)	Test Result (10 mg/L)	References
BAM-mab 01 (CBZ) *	0.32	IgG1	good	+ +	+ +	[25,37,38]
B3212M	0.15	IgG1	good	+ +	+ +	[25,39,40,41,42,43,44,45]
6C5	23.0	IgG1	poor	− −	− −	[26]
3B3	1700	IgG1	very poor	− −	− −	[26]

* In the first publication, this clone is designated “clone 1” [25].

## Supplementary Materials

The following are available online at https://www.mdpi.com/2073-4468/9/1/1/s1 and https://www.mdpi.com/2073-4468/9/1/1/s2, Description of the semi-automated data evaluation, and results of an independent screening experiment performed with commercial Nexterion E slides (Schott AG). In addition, the software for data evaluation including two sample data sets are included.
