# Supplementary figures and images for: Antibody Screening by Microarray Technology—Direct Identification of Selective High-Affinity Clones
Source: Antibodies (Basel). 2020 Jan 2;9(1):1. doi: 10.3390/antib9010001 (PMC7175374; doi:10.3390/antib9010001)

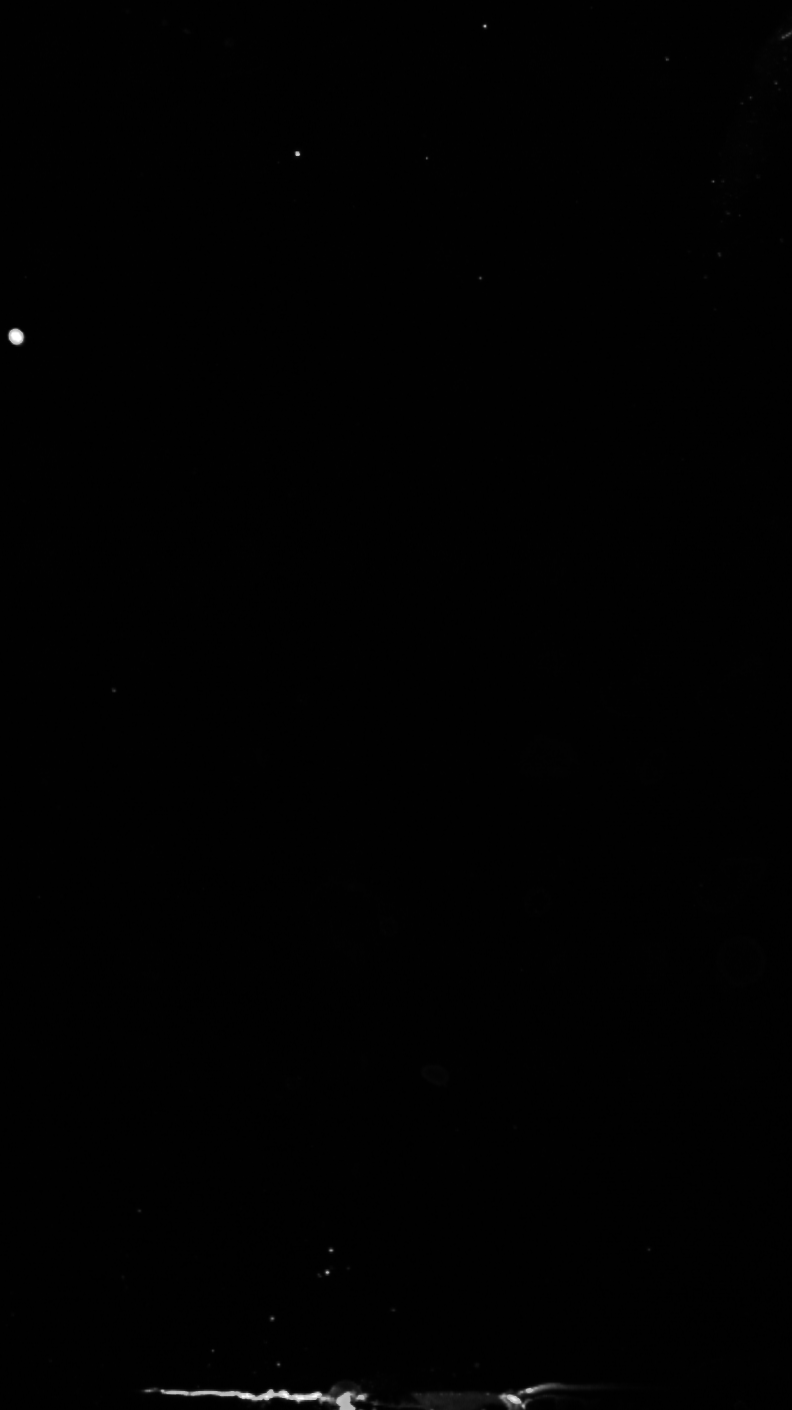

Supplement: Supplementary file 1 [file antibodies-09-00001-s001.zip › comp.tif]

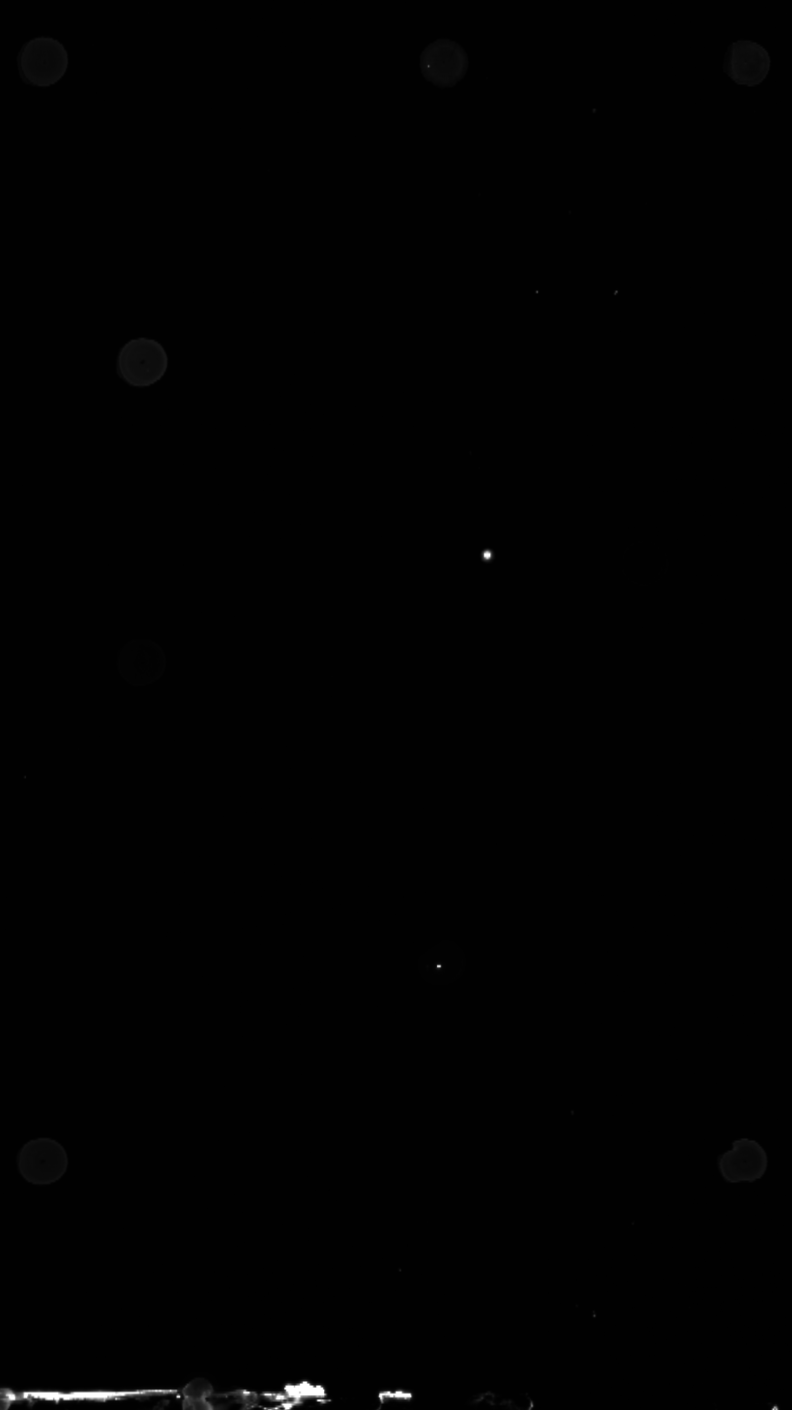

Supplement: Supplementary file 1 [file antibodies-09-00001-s001.zip › noncomp.tif]
