# Supplementary material for: Antibody Screening by Microarray Technology—Direct Identification of Selective High-Affinity Clones
Source: Antibodies (Basel). 2020 Jan 2;9(1):1. doi: 10.3390/antib9010001 (PMC7175374; doi:10.3390/antib9010001)
Supplement: Supplementary file 2 [file antibodies-09-00001-s002.pdf]

Supplementary Materials

Antibody screening by microarray technology – direct identification of selective high-affinity clones

Martin Paul [ORCID], Michael G. Weller [ORCID]*

Federal Institute for Materials Research and Testing (BAM), Division 1.5 Protein Analysis, Richard-Willstätter-Strasse 11, 12489 Berlin, Germany

* Correspondence: michael.weller@bam.de; Tel.: +49-30-8104-1150

Description of the semi-automated data evaluation

The data evaluation with Python 3.7 was performed with the development environment Spyer 3.2.2 in the Anaconda environment. The script supplied by us is intended to be used with the very application. After the files have been stored in a separate folder, the correct path needs to be copied into the script (Fig. S1)

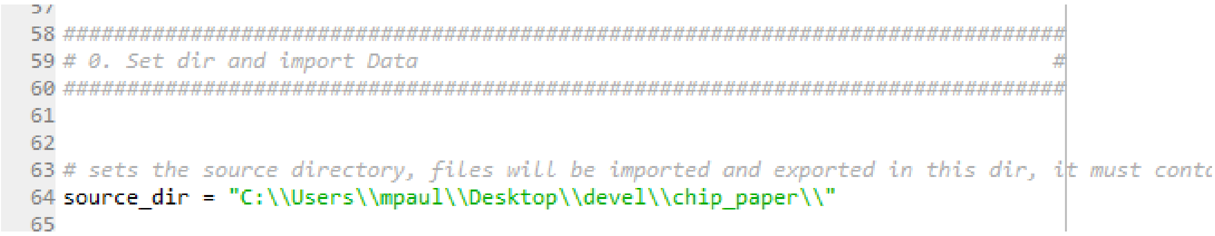

Figure S1: The correct path needs to be pasted between the " " at line 64: Please add double \\ like shown in this example.

The folder should contain the script and the competitive and non-competitive scans as .tif files. Finally, all results are exported as a .txt file in this folder. In Fig. S2, an example is shown with the path.

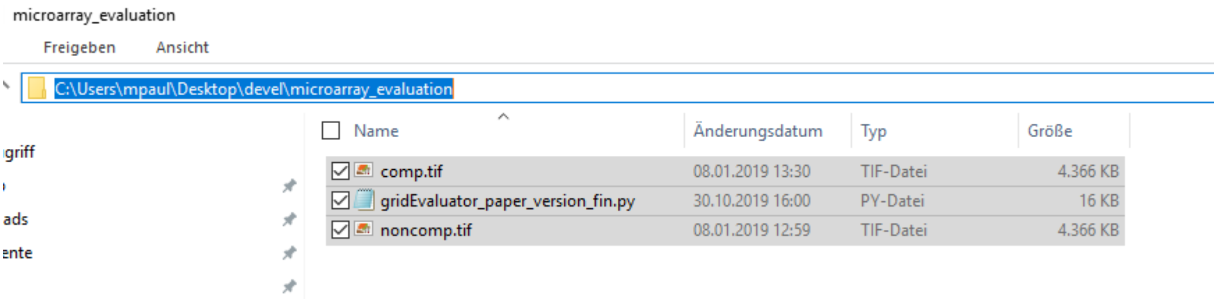

Figure S2 Example for a folder and the path.

The program requires manual user-input; the parameters needed to evaluate the given sample data of the experiment are supplied by the program as suggestions. To evaluate new scans, the central x- and y-pixel of the first upper left spot are required (Fig. S3); also the grid must be aligned with the x- and y-axis.

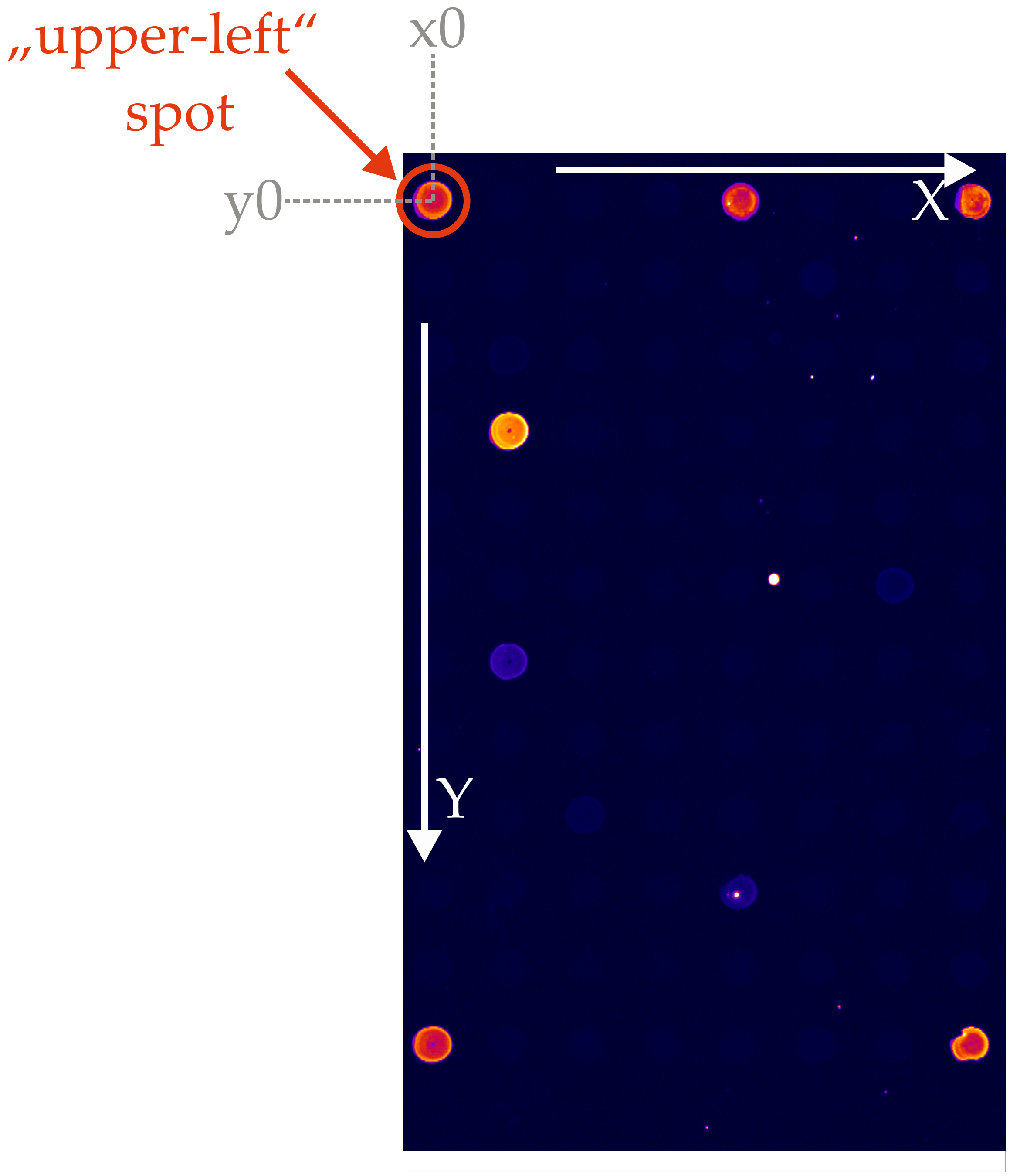

Figure S3 Determination of the upper left spot.

When the script was executed successfully, the results will be exported as three .txt files. The quotient to compare the spots along with the spot number and its coordinates on the scan are exported in the results_and_position.txt file.

Independent screening performed with commercial Nexterion E slides (Schott AG)

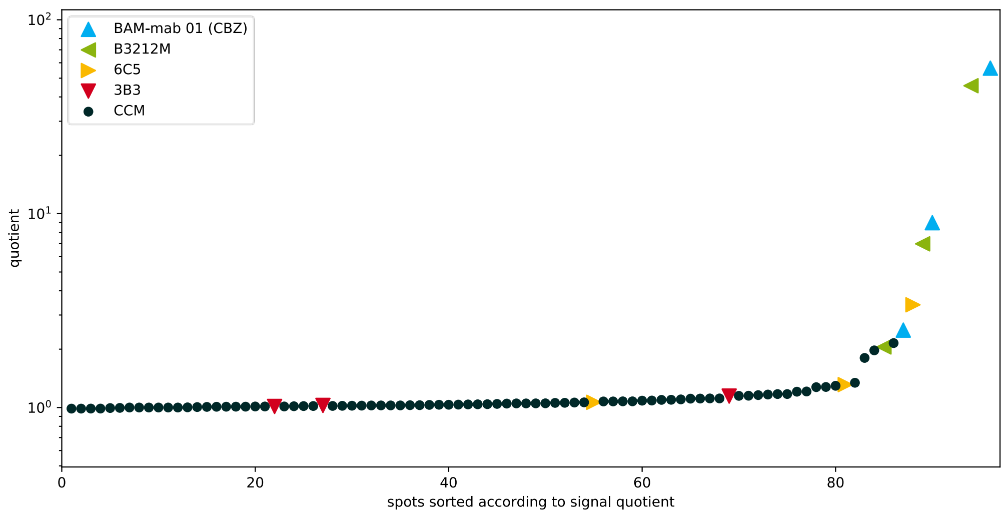

Figure S4. Signal quotients of the non-competitive and the competitive spots on the Cys-Protein-G glass slide. Known monoclonal antibodies diluted in cell culture medium are color-coded, accordingly. CCM: Cell Culture Medium (negative controls). The proven antibodies BAM-mab 01 (CBZ) and B3212M are easily identified (blue and green). The poorest antibody 3B3 (red) is not different from the blank values.

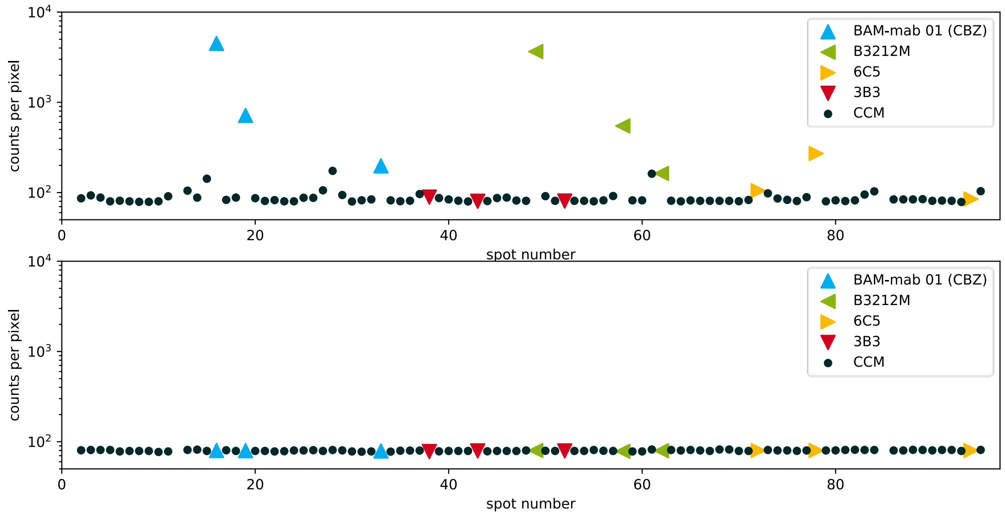

Figure S5. Signal for the spots on the Cys-Protein-G glass slide. The upper figure shows the non-competitive binding of the fluorescently labeled hapten (analyte) to the immobilized antibodies. The lower figure shows the same experiment under competitive conditions with an excess of hapten (27 mg l^{-1} analyte CBZ). Known monoclonal antibodies diluted in cell culture medium are color-coded, accordingly. CCM: Cell Culture Medium (negative controls). Blank measurements with slightly increased signals are caused by location-dependent carryover effects, which can be easily identified and eliminated. This carry-over could also be avoided by the application of a washing step during the printing.

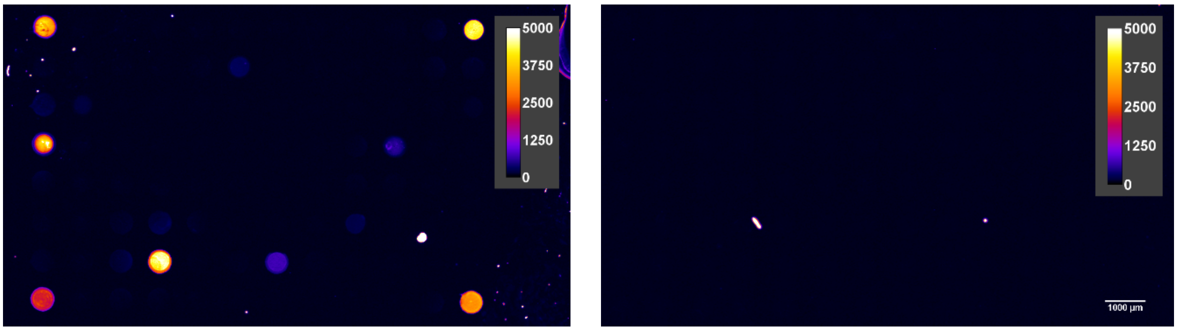

Figure S6. Non-competitive mAb incubation (left) and the competitive incubation with 27 mg L^{-1} carbamazepine (CBZ) (right). The positive spots are strongly inhibited by the hapten (analyte), which means that the respective antibodies bind selectively to the target compound CBZ.
